# User Utility Maximization in Narrowband Internet of Things for Prioritized Healthcare Applications

**DOI:** 10.3390/s22031192

**Published:** 2022-02-04

**Authors:** Nahar Sultana, Farhana Huq, Md. Abdur Razzaque, Md. Mustafizur Rahman

**Affiliations:** Green Networking Research Group, Department of Computer Science and Engineering, University of Dhaka, Dhaka 1000, Bangladesh; nahar61-2020-21@student.cse.du.ac.bd (N.S.); farhana62-2020-21@student.cse.du.ac.bd (F.H.); mustafiz@du.ac.bd (M.M.R.)

**Keywords:** NB-IoT, utility, interference mitigation, prioritized healthcare, resource allocation

## Abstract

Narrowband Internet of Things (NB-IoT) is a promising technology for healthcare applications since it reduces the latency necessary in acquiring healthcare data from patients, as well as handling remote patients. Due to the interference, limited bandwidth, and heterogeneity of generated data packets, developing a data transmission framework that offers differentiated Quality of Services (QoS) to the critical and non-critical data packets is challenging. The existing literature studies suffer from insufficient access scheduling considering heterogeneous data packets and relationship among them in healthcare applications. In this paper, we develop an optimal resource allocation framework for NB-IoT that maximizes a user’s utility through event prioritization, rate enhancement, and interference mitigation. The proposed Priority Aware Utility Maximization (PAUM) system also ensures weighted fair access to resources. The suggested system outperforms the state-of-the-art works significantly in terms of utility, delay, and fair resource distribution, according to the findings of the performance analysis performed in NS-3.

## 1. Introduction

Establishing smart hospitals using suitable technologies is a necessity of time, and it requires an appropriate replacement of the physical interaction between patients and doctors by a reliable virtual one [1]. In a smart healthcare environment, the utility of a patient quantifies how fast and how reliably his/her real-time physical condition is reported to a central healthcare information management system. Moreover, different health parameters are typically interdependent, and they have different importance levels. Thus, the problem of user utility maximization is translated as a multi-objective data delivery performance enhancement problem. Furthermore, it requires the prioritization of critical data over normal or regular event reporting. For example, when a patient’s extreme high/low blood pressure (BP) is considered as the most important health parameter, the corresponding blood sugar level and respiratory rate become relatively important and a few others as regular health parameters.

Healthcare is boosted as an application domain that appears to be very promising for enhancing service quality of the patients due to the Internet of Things (IoT), cloud computing, and Wireless Body Area Network (WBAN) technologies, as well as the interactions among those [2]. Some wireless technologies (e.g., ZigBee, Bluetooth, LoRa, Sigfox) are used in healthcare applications; however, some recent studies [3,4] have already revealed that the Narrow Band IoT (NB-IoT) is more suitable for healthcare applications in terms of licensing policy, long-range data transmission performance, energy-efficiency, etc., than other technologies. The fact that the NB-IoT is a Low Power Wide Area Network (LPWAN) technology facilitating a long-range and deep indoor coverage makes it perfect for a typical smart hospital context [5,6]. Furthermore, it offers seamless connectivity between patients and concerned medical personnel.

The Narrow Band Internet of Things ( NB-IoT) is a radio access technology developed by the third-generation partnership project (3GPP) that turns out to be categorically prevalent for real-time sensing and monitoring applications [5], including smart metering, connected personal appliances, smart hospitals, etc. [6,7]. Licensed frequencies of today’s cellular network can provide NB-IoT devices more accurate traffic management, high-quality service (QoS) assurance over broad areas, and higher reliability [1]. Interference mitigation in NB-IoT becomes another challenge as several transmissions frequently occur at the same time [5] in a typical healthcare application; otherwise, it might hamper reliable and guaranteed data delivery. Solving such a multi-objective data delivery performance enhancement problem that requires data prioritization becomes more critical in a resource-constrained environment.

In [8], the authors developed an optimization algorithm, named bRRM, which analyzes inter-carrier interference for radio resource management for OFDM (Orthogonal Frequency Division Multiplexing). Their proposed resource allocation strategy boosts up throughput; however, event relationship, queue management, etc., parameters were not incorporated. In [9], the authors conducted an experiment, collecting the continuous data of a patient from a weight scale based on NB-IoT technology, which can be sent to a specific server at any time. The system was developed to reduce unnecessary data storage operations by transferring healthcare data from medical instruments to a web server utilizing NB-IoT. Their system lacks in developing an application that can separate and identify patient data, which resembles event categorization and inter-dependency of health parameters, respectively. The problem of data collection using NB-IoT devices in a smart hospital was first addressed in [10]; their inadequate policies of the given wireless protocols merely handled the collection of huge data from numerous NB-IoT devices in a smart hospital.

The authors of [11] investigated the realistic performance of NB-IoT in a healthcare monitoring system in terms of effective throughput and latency. However, the work did not optimize the required throughput, delay, and device density jointly. The authors mainly focused on the application-specific analysis of cell throughput, device capacity, and latency of NB-IoT, keeping aside joint optimization and radio resource allocation with prioritized scheduling. Later, in [12], an intelligent radio resource management algorithm, iRRM for NB-IoT, was developed with efficient resource allocation considering inter-cell interference (ICI), coverage extension, and the repetition factor. In a healthcare environment, due to the periodic transmission of sensory data, a higher density of User Equipment (UE) always incurs higher delays. However, the necessity of prioritizing interdependent health parameters needs to be addressed to reduce the delay, and it can be implemented through traffic classification. Moreover, achieving expected user utility under the interference-aware radio resource allocation needs further investigation [1,9].

This paper develops an interference-aware radio resource allocation for NB-IoT devices in healthcare applications that prioritizes data traffic from different sources and maximizes user utility. We define user utility as a joint metric of achievable data rate and event influenced priority, where users communicate data to the base station (eNB) minimizing interference. The proposed resource allocation is optimized according to the interdependent event under the same time slot with the intention to maximize the overall utility of all users that introduces Priority Aware Utility Maximization (PAUM). This model is projected to achieve the increased performance for healthcare applications. The key contributions of this work are summarized as follows:

- We design a Priority Aware Utility Maximization (PAUM) system for providing high-quality medical care in such a way that is convenient for patients so that data transmission can occur according to the application’s urgency level.
- We develop an event-influenced prioritized access scheduling of critical and non-critical data packets exploiting Bayes Theorem, which determines the importance of events in a probabilistic distribution.
- Under certain constraints, the problem of maximizing user utility has been formulated as a multi-objective non-integer linear programming (MNILP) problem that ensures high performance and fair resource allocation to the users.
- The performances of the proposed system have been carried out in Network Simulator version 3, and significant performance improvements are observed for various events and prioritized users in healthcare applications.

This paper is organized as follows: Section 2 presents relevant studies, Section 3 defines the system model and assumptions, Section 4 elaborates the design components of the PAUM system, and Section 5 analyzes the performance results. Finally, Section 6 contains the conclusion.

## 2. Related Works

According to various studies, the healthcare industry utilized radio communication technologies in a variety of ways as it approached advancements in care and rehabilitation. The Wireless Body Sensor Network (WBSN) was one of the most promising concepts; it consisted of a network of sensor nodes worn on the body, capable of capturing, analyzing, and wirelessly transmitting one or more types of physiological or environmental information [13]. Wireless Body Area Network (WBAN), also known as a body sensor network (WBSN), allowed for health monitoring everywhere on the human body at any time [14]. On the other hand, IEEE 802.15.6 provided low-power, short-range, and extremely dependable wireless communication within the body’s surrounding area [15]. For short-, medium-, and long-range communications, WBSNs could communicate with other networks by a number of wireless protocols such as ZigBee, Bluetooth, Ultra-Wide Band, Wireless LAN (Wi-Fi), WiMAX, etc. [15]. Their uncertainty of battery lifetime and short range communication are the major disadvantages [13], which prompted the introduction of low-power, wide-area network technology (LPWAN) for healthcare applications, such as LoRa, Sigfox, and NB-IoT. According to [2,6], while the unlicensed LoRa and Sigfox technologies focused only on supporting low-cost applications in an energy-efficient way, the NB-IoT is directed to augment high QoS and low latency.

The problem of maximizing utility considering rate maximization or interference mitigation or access prioritization to the application in an NB-IoT based healthcare system has been categorized into two groups. These are: the basic operation of NB-IoT and resource management in NB-IoT-based healthcare applications. This section epitomizes state-of-the-art papers covering both of these groups.

A real-time application of a patients’ health status monitoring system based on theIoT was discussed in [1] with a brief overview. This work aimed to automatically provide suggestions to the patient according to his health condition. According to their analysis, a licensed frequency supported cellular network (e.g., NB-IoT) ensures reliable and efficient communication in the healthcare system. An intelligent medical plan was based on NB-IoT technology and a smart hospital information management system was developed and explored in [4].

The study in [5] explored the aspect of the physical and medium access control (MAC) layer issues of NB-IoT in detail. The authors elaborately discuss synchronous and asynchronous networks elongated with the 5G network. They derive theories for resource allocation, link adaptation, coverage, and capacity issues for specific NB-IoT-based applications, such as healthcare, smart meters, smart grids, etc. Furthermore, a continual survey of Low Power Wide Area Network (LPWAN) technologies of LoRa and NB-IoT in [6] is also discussed broadly with their basic specification for different services such as IoT industries (Smart Agriculture), public services (Smart metering), personal services (healthcare), etc. The long-range (LoRa), as an unlicensed LPWAN technology, has some advantages in battery lifetime, cost, and capacity, whereas the licensed NB-IoT technology offers efficiency in terms of QoS, latency, reliability, and coverage range. They compared and described the technical differences of LoRa and NB-IoT, where the key aspects consist of physical features, network architecture, MAC evaluation, several IoT applications, QoS, lifetime, and latency. In the paper, IoT cases studies were widely categorized as *IoT personal*, *IoT public*, *IoT industries*, and *IoT appliances*. Additionally, it focused on how the network deployment for various applications such as smart agriculture, smart metering, and smart healthcare using NB-IoT will deliver future low-cost services in hard-to-reach areas. This paper also presented the cases of successful implementations of diverse applications in different countries, mainly in Korea, Japan, and China.

In [9], the authors experimented with collecting the continuous data of a patient to a specific server from a weight scale based on NB-IoT technology. By transferring healthcare data from medical instruments to a web server using NB-IoT, the system aimed at decreasing unnecessary data storage operations. However, it certainly lacks in developing an application that could differentiate and identify patient data classification.

To support the healthcare system, the introduction of connecting intelligent devices to smart hospitals using NB-IoT was discussed in [10], as NB-IoT provided a new way for connecting devices to meet the requirements of small-scale data over a long period of time. They also overviewed the application scenarios and characteristics of medical IoT devices in smart hospitals. Here, high latency and poor mobility influenced the undesirable effects of NB-IoT. The authors briefly discussed the challenges and future directions of building a smart hospital using NB-IoT. They mentioned the following challenges: (i) the accuracy and reliability of the data, (ii) security and privacy, (iii) wireless communication interference, (iv) energy consumption of terminals, and (v) NB-IoT performance testing, i.e., frame structure, resource allocation methods, connection configuration, etc. A remote healthcare monitoring system using NB-IoT is proposed in [11]. NB-IoT is an emerging technology that provides low-cost, long-range, and low data rate coverage extension in delay-tolerant applications. The paper analyzed the realistic performance of healthcare data using NB-IoT in terms of effective throughput and latency with various modes of operation such as in-band and stand-alone deployment.

In [16,17,18], NB-IoT evolutions, technologies, and open issues were narrated briefly for their suitable applications. They opened it as the newest Long Term Evolution (LTE) by 3GPP and one of the LPWAN solutions to achieve super coverage, low power, low cost, and massive connection, which was very likely to be used by a smart hospital system. Other than healthcare, a comprehensive discussion in [19,20,21,22] presented the difficulties of achieving high QoS for smart grid connection for unlicensed LPWAN technology. They proposed NB-IoT for smart grid connection, where the data rate, latency, range, etc., were discussed to satisfy all requirements of QoS.

A systematic analysis of NB-IoT’s Quality of Service in [21,22,23,24,25,26] approached the uplink transfer of individual sensor data from a single sensor and the downlink transfer of individual commands. The authors analyzed relevant physical- and application-layer QoS parameters and contributing factors in a real NB-IoT network, which was studied in Germany for the first time. A detailed evaluation of the end-user QoS of NB-IoT is presented in the paper. In [8], the optimized radio resource management for OFDM (Orthogonal Frequency Division Multiplexing) was described, which is referred to as bRRM and which solely considers inter-carrier interference. The basic signal format used in 4G LTE (i.e., NB-IoT) is OFDM, which was suited for high-speed data transmission because it resisted narrowband fading caused by reflections and the general propagation qualities at these frequencies by using several carriers, which carried a low data rate. Radio resource management was a vital issue for the OFDM system. The authors devised a resource allocation technique that improved system performance while increasing throughput. Prior to resource management, they failed to recognize the relevance of cell element organization, which is comprehensively discussed in our work. We also looked at NB-IoT, a licensed LPWAN based on the OFDM method that featured to make it ideal for healthcare applications.

The way in which radio resources can be efficiently managed in the NB-IoT context to permit large IoT devices is proposed in [12] regarded as iRRM. Repetition was a vital aspect to incorporate with the resource allocation strategy in order to achieve coverage augmentation. Initially, the researchers looked at single-cell achievable data rates, determining a trade-off between information rates, latency, and supported devices. Aiming to improve the system sum rate, they proposed NB-IoT-based QoS-aware resource allocation in a multi-cell situation. They created an optimization framework as well as a non-optimal solution for a large number of users in this regard. Finally, the authors developed a cooperative game strategy to achieve a certain system performance and evaluate their concept with evidence-based findings. Their findings revealed that providing higher data rates with NB-IoT comes at a cost, including fewer supported devices and increased delays. This paper focused on jointly optimizing the data rate, delays, device density, and resource allocation to achieve a better solution using NB-IoT. Moreover, in the case of healthcare applications, data sizes are small, requiring less power and long-distance communication, both of which are well supported by NB-IoT.

In this paper, we emphasize on resource allocation methods for smart healthcare system using NB-IoT addressing parameters that were not reflected in previous works. This paper discovers the idea of event categorization to identify an appropriate user for data transmission in a certain time slot for the allocation of radio resources. We explore the factors that affect event-influenced access priority in the NB-IoT healthcare environment and formulate an optimization framework to maximize user utility in networks along with some constraints. In this proposed model, prioritized users (uplink cases) communicate with the base station with interference-aware radio resource allocation while considering the appropriate users, data rate, time slot, and QoS constraints. Such a model is expected to offer higher performance for healthcare applications.

## 3. System Model and Assumption

This paper addresses the problem of developing an efficient healthcare monitoring system using NB-IoT that necessitates access prioritization of UEs to communicate with others through a base station (eNB) and proficient channel allocation with different time slots. This section presents the network environment and application model followed by assumptions.

### 3.1. NB-IoT Network Environment and Application Model

The NB-IoT is an LTE variant, especially in Internet of Things (IoT)-specific networks. The main mechanism of the NB-IoT network is the evolved packet system (EPS), which employs two types of optimizations: user plane and control plane EPS optimization [6,27].

**Figure 1:**
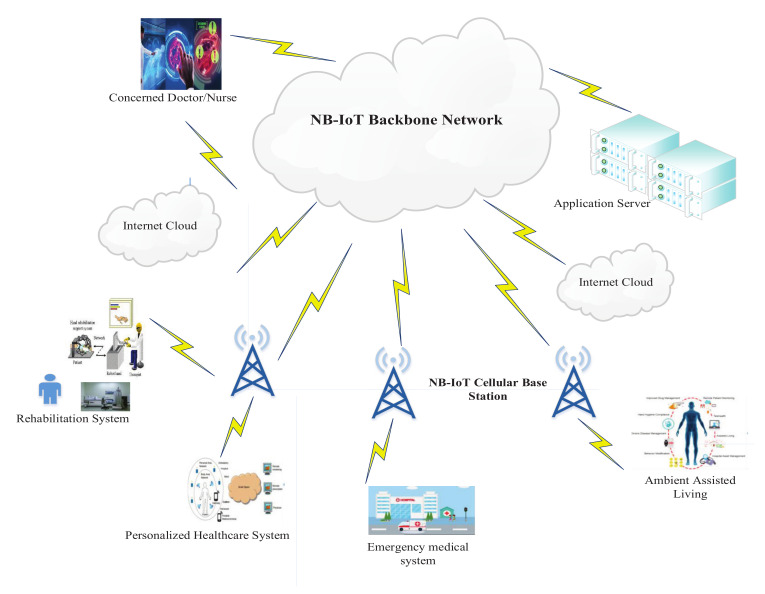
A smart healthcare application architecture using NB-IoT.

We assume that NB-IoT devices are installed in a hospital environment that generates specific sensed data from the operation theatre, patient ward/cabin, or doctor’s chamber (OPD) and sends them to the central server through eNB. Figure 1 represents the system architecture of the NB-IoT-based network, which comprises a set of eNBs connected with various healthcare monitoring applications, including remote patient monitoring, personalized healthcare systems, rehabilitation systems, ambient assisted living, emergency medical systems, and telemedicine systems [8,12].These applications use various sensors such as temperature, pressure, motion, image, etc., where smart UEs such as watches, weight scales, blood sugar and blood pressure sensors, gastric stimulators, pulse oxymeters, etc., are very likely involved in healthcare applications.

As small data volumes need to be infrequently transmitted in an NB-IoT environment, there must be some scheduling for UEs. Various UEs generate diverse data types, which demand the necessity of maintaining various priorities p∈P among them based on the urgency. The communication of uplink users is considered in this model, and usually the NB-IoT uplink transmission occupies 180 kHz of bandwidth and supports two subcarriers spacing, 3.75 kHz and 15 kHz. However, this network model explicitly investigates the feasibility and effectiveness of the event-influenced prioritized access of UEs under good resources to maximize user utility.

Certain essential issues are concerned to explore the healthcare applications using cellular technologies such as scheduling the access of IoT devices [8,11] from the environment, then data transmission and management inside the smart hospital environment to communicate with the concerned area. At the same time, interference minimization is also an important issue, as a large number of individuals are expected to connect with the smart hospital system.

### 3.2. Assumptions

We assume the data transmission between an eNB and an uplink user z∈Z follows a SC-FDMA( Single carrier frequency division multiple access) signal, which follows LP-OFDMA (Linearly precoded orthogonal frequency division multiplexing). In this system, a single channel c∈C is modulated by multiple sub-carriers, and different time slots t∈T of single a time frame (T) are mapped with those sub-carriers. Here, the eNB allocates each uplink user z∈Z a particular time slot t∈T according to its priority p∈P in a single channel c∈C. An uplink user z∈Z can enjoy the maximum peak data rate $r_{z}^{max}$ (66kbps) through a channel using a one-time slot. The notations used in this paper are summarized in Table 1.

## 4. Design of the PAUM System

This section characterizes previously discussed research challenges into achievable clarification. Addressing proper access among UEs to communicate with eNB, the events classification model opens up the idea of handling the access based on the priorities of different events.

**Figure 2:**
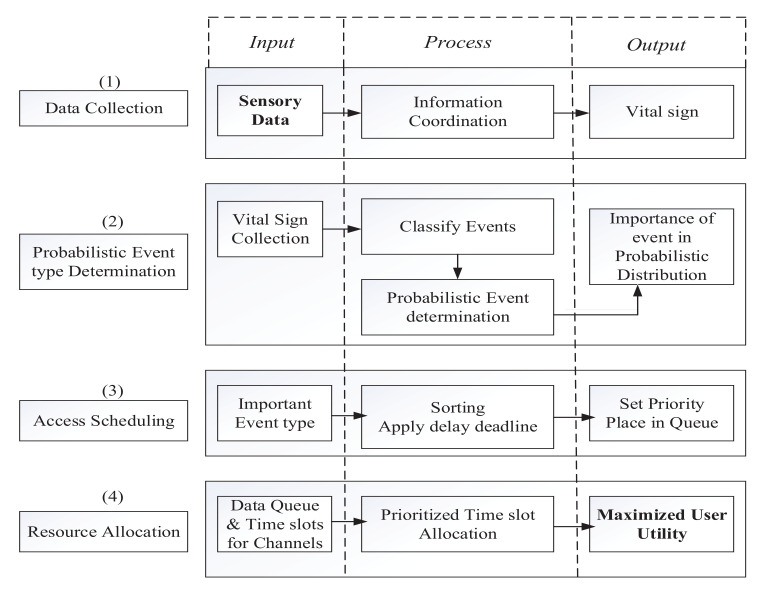
Functional Block Diagram of the Proposed PAUM System.

The block diagram in Figure 2 illustrates a detailed understanding of our proposed interdependent event influenced prioritized data transmission access scheduling. The following subsections will delineate the events classification and dispensation of proposed algorithms. Preparing a scheduling of UEs tends to mitigate any mutual interference. Maximizing user utility through the optimal allocation of bandwidth resources has been depicted in the latter part of this section.

### 4.1. Events Classification

It is significant to ensure the data transmission of the healthcare system by making prioritization for various data from different sensors and to guarantee the diverse quality of services to those events [28]. In this paper, the prioritized scheduling of data packets generated from different UEs is inspired by the fact that different diagnostic reports are related to each other, and the importance of an event is determined by the degree of its relevance with one or more other parameters. Initially, the eNB allocates time slots to uplink users, assuming that they are producing data within the normal range. Once it receives data from different users, it examines and finds out the relationship among the events, including the selection of the priority of the concerned event.

Table 2 gives some examples of the event classification using diseases, probable locations to collect data, and the corresponding diagnosis. First of all, we attempt to reference the syndromes and analogous diagnoses to categorize the events. The instantaneous identification of any disease inside a smart hospital is reflected as a Critical Event (CE), which is required to be handled as crisis/emergency. The diagnoses and treatments that are applied as a direct intervention or in a supportive role for a critical event are termed as Relevant Events (RE). Then, all unvarying procedures are named Normal Events (NE). There are numerous diseases with their own symptoms and diagnoses. Still, some of the diseases and their diagnoses that can be applied with smart-sensor-based equipment are mentioned in the proposed Table 2. By all means, CE must get a chance first to transmit data with the highest priority, followed by RE and NE.

Applying the Bayes Theorem [29], the sensed information is categorized into different types of events, based on its vital sign for effective data transmission. This proposed system uses the concept of a candidate model that approximates a target function for mapping inputs to outputs known as a hypothesis. The learner considers these inputs as some set of candidate hypothesis *s* and is interested in finding the most probable hypothesis s∈S given observed data *R*, which returns the output as the categorized event described in Algorithm 1 in the following subsection.

According to Algorithm 1, event classification depends on sensory data and their co-relation. We use sensory data to determine event-wise training data based on its threshold value. This training data act as prior knowledge to find the likelihood of events in our proposed application. The Bayes Theorem expresses the likelihood of an event based on prior knowledge of the conditions that may be associated with it. Therefore, the Bayes Theorem can be relied on to identify the likelihood and categorize the occurrences with specific conditions to determine the more accurate probability of the emerging event.
**Algorithm 1** Probabilistic determination of Event Type.**Input**: N,E,V,S: Sets of UEs, events, vital signs, and hypotheses, respectively$V_{th}^{low},V_{th}^{high}$: Lower and higher thresholds of vital sign**Output**: Event Determination Probability and Maximum probable hypothesis1:**for** each n∈N **do**2: **if** ($V_{n}<V_{th}^{low}\left|\right|V_{n}>V_{th}^{high}$) **then**3:  $\partial_{C}\leftarrow V_{n}$;4: **else**5:  **if** ($C_{v}\to R_{v}$ returns true) **then**6:    $\partial_{R}\leftarrow V_{n}$;7:  **else**8:    $\partial_{N}\leftarrow V_{n}$;9:  **end if**10: **end if**11:**end for**12:**for** each n∈N **do**12: $P\left(s_{n}|\partial_{C}\right)=\frac{P\left(\partial_{C}|s_{n}\right)P\left(s_{n}\right)}{P\partial_{C}}$; $P\left(s_{n}|\partial_{R}\right)=\frac{P\left(\partial_{R}|s_{n}\right)P\left(s_{n}\right)}{P\partial_{R}}$; $P\left(s_{n}|\partial_{N}\right)=\frac{P\left(\partial_{N}|s_{n}\right)P\left(s_{n}\right)}{P\partial_{N}}$;13:**end for**14:**for** each $E\in \{\partial_{C},\partial_{R},\partial_{N}\}$ **do**15: **for** each n∈N **do**15:  $s_{n}=\underset{\forall s\in S}{\arg\max}\left(P(s|E)\right)$;16: **end for**17:**end for**18:**return**$s_{n}$;

### 4.2. Event Influenced Prioritized Data Transmission Access Scheduling

In the proposed PAUM system, algorithms and optimization functions are executed in the eNBs. After determining event types, an eNB sends specific transmission slots to the UEs according to their event priorities so as to achieve maximum utility. Individual data sources follow the eNB for sending sensory data. In this section, we formulate two consecutive algorithms intended for an event-influenced priority scheduling to be executed in the eNBs.

The Algorithm 1 generates a probabilistic event recognition output for a single channel based on gathered vital signs in a specific time window. A vital sign is defined as a sign that aids in the detection or monitoring of medical disorders or the measuring of a live organism’s physiological function [30]. Vital signs are proof of the body’s current physical functioning, providing important information such as blood pressure, pulse rate, breathing rate, etc., to determine the urgency. The Bayes Theorem [29], which is based on hypothesis and associated individual training data, was used to determine the most likely event type (Critical, Relevant, or Normal). In this algorithm, *N* is the set of UEs, *V* is the set of vital signs, and *S* is the set of hypotheses used as input. In lines 1–11, the vital sign readings and their thresholds are exploited to categorize traffic types—critical training data $\partial_{C}$, relevant training data $\partial_{R}$, and ordinary training data $\partial_{N}$, having priorities 1, 2, and 3, respectively, where the lower number represents higher priority.

Note that the relevant training data are revealed by combining two propositions—$C_{v}$: $V_{n}^{C}$ is a critical vital sign; and $R_{v}$: $V_{n}$ is a supporting vital sign for any critical event, $V_{n}^{C}$. The implication $C_{v}\to R_{v}$ denotes whether a critical event needs the support of another vital sign. If the supporting vital sign is required for the associated critical event of that particular moment, it is counted as relevant training data. It then employs Bayesian learning to determine the probability of an occurrence based on new data that is connected to the event in lines 12 to 13. In fact, Bayesian approaches can incorporate probabilistic prediction hypotheses. In PAUM, the hypothesis is a vector of six constraints, specifying the values of the six attributes of vital sign, location{cabin, OPD, Emergency, Remote}, range, forecast, decision, and type, and combines training examples for the target concept “Event type”. Finally, the algorithm searches the maximally probable hypothesis by MAP (Maximum a posteriori) hypothesis, which returns the most probable event determination in lines 14 to 17.

Then, Algorithm 1’s outputs are received by Algorithm 2 as ${\overset{´}{s}}_{n}$—the sorted probabilistic determination of events. Another input is $d_{n}$, the UEs’ delay deadline for individual positioned events. Initially, the priority value is inserted in $P_{n}$ applying the $n^{th}$ positioned UEs vital sign record using function PRIORITY($V_{n}$) on line 2, and that UE is placed in a subsequent Queue position. Then, in lines 3–4, the priorities and queue positions are updated depending on the delay deadline values of two consecutive events given that they have a very small difference (ϵ) in their $\overset{´}{s}$ values. It assists us in assuring that important packets are transmitted earlier than others. Finally, sorted priority events are stored in a queue as a result of Algorithm 2.
**Algorithm 2** Prioritized Data Transmission Queue Scheduling Algorithm.**Input:**${\overset{´}{s}}_{n}\leftarrow s_{n}$ sorted in descending order. d: Set of delay-deadlines of all UEs**Output:***P*: Set of Priorities, *Q*: Transmission Queue**Initialization**: counting variable, t← 11:**for** each n∈N **do**2: $P_{n}\leftarrow$ PRIORITY($V_{n}$);  $Q_{t}\leftarrow n$;3: **if** $\left(|{\overset{´}{s}}_{n}-{\overset{´}{s}}_{n+1}|\leq \epsilon \;\&\&\;d_{n}>d_{n+1}\right)$ **then**4:  $P_{n}\leftarrow$ PRIORITY($V_{n+1}$);  $Q_{t}\leftarrow n$;  t←t+1;5: **else**5:  t←t+1;6: **end if**7:**end for**8:**return**Q,P;

Let us look at an example situation in which three vital signs are used to promote a better understanding of algorithms. Assume a smart BP (Blood Pressure) sensor sends data in a single channel from multiple places (OPD, Emergency, Ward, Cabin) during a time slot, t∈T, such as $V_{t}^{BP}$ = {(80, 120), (45, 120), (82, 115), (80,185)}. A smart blood sugar monitor collects data $V_{t}^{BS}$ = {4.2, 5.6, 6.7, 7.5} and a smart pulse oximeter collects data $V_{t}^{PO}$ = { 97, 96.5, 98, 99} in the same time frame and channel. The Bayes Theorem thus aids in assessing the likelihood of a forthcoming event based on its vital sign record collection across multiple sites combine as a hypothesis. Both the second and fourth BP recordings in our example exceed the threshold, suggesting they are critical events. A chest X-ray/ECG is a significant relevant diagnosis in this circumstance to support the identified critical event. Then, both blood sugar and oxygen saturation levels are within acceptable limits, indicating that the occurrence is normal. Finally, the UE for blood pressure is given top priority, *P*, and is positioned first in Queue, followed by the UEs for chest X-ray, blood sugar, and pulse-oximeter. This is how the eNB categorizes the UEs according to the event.

#### Multilevel Queue with Preemptive Round Robin Scheduling

Algorithm 2 returns the transmission queue result, which reflects the data transmission priority. Because this queue uses a preemptive RR (Round Robin) algorithm, transmission begins with a higher priority data packet and ends with a lower priority. We examine the suggested method in this part, taking into account changes in location inside the smart hospital. As a result, we investigate how other UEs obtain a schedule from the base station during the transmission of a queue that has already been scheduled.

**Figure 3:**
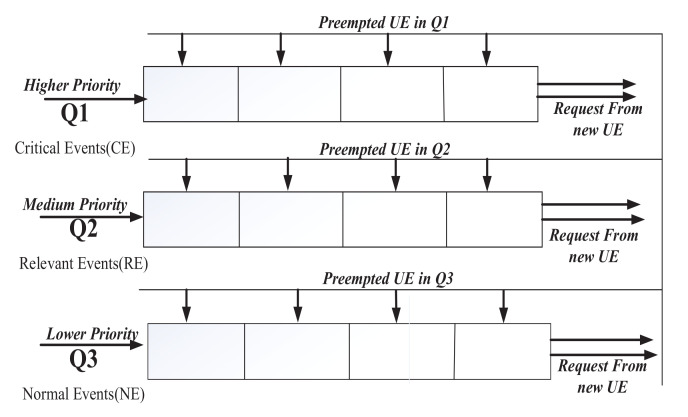
Preemptive multi-level priority queue scheduling.

If any unscheduled higher priority events desire to join the eNB during the current transmission queue process, they must be handled by an eNB for effective scheduling and prioritized access. In this case, Figure 3 shows that the ready queue is divided into a multilevel queue approach, with the first queue Q1 having the highest priority and containing all critical events, the second queue Q2 having the next higher priority and representing all relative events, and the third queue Q3 having a lower priority and holding normal events.

Preemptive queue scheduling assigns a spot in Q1, Q2, or Q3 according to the priority of any new UE joining this access scheduling mechanism. When multilevel queuing is used, there is a significant risk of starvation [28]. To mitigate data transmission starvation, the aging algorithm [31] is used.

### 4.3. Interference Mitigation

The next research concern is interference mitigation among UEs while communicating with the base station. The frequency band allocation for NB-IoT in standalone mode or in-band mode may not be simultaneous in all cells, resulting in inter-cell interference (ICI) [7,11]. Any possible noise/interference may cause natural readings to be disrupted, resulting in incorrect identification and treatment [10]. As a result, an effective strategy for eliminating interference and providing QoS to users must be developed.

The UEs are assigned to a transmission queue to prioritize event-influenced access to the base station. Currently, the system is working on making the most efficient use of available resources while addressing interference mitigation. For an OFDMA (Orthogonal Frequency Division Multiplexing)-based cellular network, where cells are separated into discrete areas with different frequencies, frequency reuse methods are the best interference management strategies. The signal to interference and noise ratio (SINR) is the most essential factor in determining the amount of inter-cell interference and evaluating the interference management technique’s performance.

The SINR formula, in general, is as follows:

(1) $$SINR=\frac{\rho \times h}{I_{z}+N_{0}}.$$

Here, ρ is the received power of the desired user, *I* is the total interference, *h* is the fading channel gain, and $N_{0}$ is Additive white Gaussian Noise.

Now, we can define, the interference:

(2) $$I_{z}=\sum_{k\in B}\rho_{z}^{k}\times h_{z}^{k}.$$

Equation (2) implies that $I_{z}$ is the inter-cell interference received by the user *i* of Base station *b* in the uplink transmission scheme from the users of neighboring Base stations using the same resource, where $\rho_{z}^{k}$ is the transmit power from *z* the downlink/uplink user of neighboring cell *k*, $h_{z}^{k}$ is the channel gain between the neighboring BS *k*, and *j* is the uplink user of Base station *b*.

The data rate in Equation (3) can be defined for uplink users as follows:

(3) $$r_{z}^{b}=\log_{2}\left(1+SINR_{z}^{b}\right).$$

### 4.4. User Utility Maximization

This section delineates user utility and develops an optimization framework to optimally allocate resources based on the desired user utility. We want to allocate bandwidth resources to the UEs so that the user data rate is maximized, the interference is minimized, and the high-priority users obtain access to good quality resources.

Thus, the Utility function $U_{z}$ for an *uplink user,* z∈Z is defined as:

(4) $$U_{z}=\frac{r_{z}}{P_{z}XI_{z}};\;\;\forall z\in Z$$

and the optimization framework is formulated as:

(5) $$Maximize:f=\sum_{\forall t\in T}\sum_{\forall z\in Z}\sum_{\forall c\in C}i_{z,t}\times U_{z}\;$$

Subject to:

(6) $$i_{z,t}^{c}\in \left\{0,1\right\};\;\;\forall t\in T,\;\forall z\in Z,\;\forall c\in C$$

(7) $$r_{z,t}^{c}\leq r_{z,max};\;\;\forall t\in T,\;\forall z\in Z,\;\forall c\in C$$

(8) $$\sum_{t\in T}k_{z,t}^{c}=1;\;\;\forall z\in Z,\;N<T,\;\forall c\in C$$

(9) $$\sum_{t\in T}k_{z,t}^{c}\leq 1;\;\;\forall z\in Z,\;N>T,\;\forall c\in C$$

(10) $$\rho_{z,t}^{c}\leq \rho_{max};\;\;\forall t\in T,\;\forall z\in Z,\;\forall c\in C$$

(11) $$h_{z,t}^{c}\geq \;h_{min};\;\;\forall t\in T,\;\forall z\in Z,\;\forall c\in C$$

Here, the objective function in Equation (5) is formulated as an MNILP (Multiobject Non-Integer Linear Programming) to be solved by the eNB. Maximizing utility with an allocated channel and its time slot for all uplink users resulted in an optimum solution with some effective constraints.

*Allocation Constraint*: The binary variable $i_{z,t}^{c}$ in Equation (6) contains 1 if an uplink user z∈Z is allocated a time slot t∈T of channel c∈C, and 0 otherwise.

*Budget Constraint*: The measurement variable $r_{z,t}^{c}$ in Equation (7) enumerates how much data of a UE is possible to be transmitted through a particular channel c∈C with an allocated time slot.

*Time Slot Allocation Constraint*: The constraint in Equations (8) and (9) creates a restriction that exactly one time slot in a time frame can be allocated to an uplink user *z* if the number of users is less than the available time slots; otherwise, the allocation of a time slot to an uplink user *z* is not guaranteed but is rather opportunistic following its priority.

*Interference Constraint*: Constraints in Equations (10) and (11) mitigate interference issues by keeping power within a certain range while choosing a channel that offers gain above a certain minimum threshold. In the PAUM system, the utility of a patient emphasized the QoS of a successful and reliable smart healthcare system. The maximization of utility for any uplink user upgrades the system’s access reliability; under the time slot assignment, its higher priority and its higher data rate and event-influenced delay is achieved.

## 5. Performance Evaluation

This section presents the comparative performances of the proposed Priority Aware Utility Maximization (PAUM), Basic Radio Resource Management (bRRM) [8], and Intelligent Radio Resource Management (iRRM) [12] with diversified parameters in network simulator version-3 [NS-3] [32,33] using a typical LTE network cell for a smart healthcare system.

### 5.1. Simulation Environment

Table 3 represents the simulation parameters and their values following 3GPP standards [34], where the cellular layout is considered as a hexagonal grid using three sectors per site with an inter-site distance of 500 m along with a frequency band of 900 MHz considering a 180 kHz channel bandwidth. The system-level performance is developed by an NS-3-based LTE environment, where multiple UEs are connected with multiple eNBs, supporting up to 10 to 100 eNBs and 100 to 1000 UEs with good channelization specifying the time slot. The various NB-IoT devices installed in a typical hospital environment, including the OPD, Ward, Emergency, Cabin, and Operation Theater, have been considered as data generation sources in the simulation environment. Here, we consider three types of events, namely, critical, relevant, and normal. We also consider the occurrence of an event and its type in a certain NB-IoT UE using uniform random distribution. Therefore, in a given scenario, the number of events varies from 0 to the total number of UEs intended for communication, each generating 3 to 15 packets/sec randomly. The data ranges and payload information sizes produced from different sensors are adapted from [11] and resemble a practical healthcare application environment. The RadiobearerStatCalculator is used as a simulation trace file to obtain a view of different parameters. The experimental data are analyzed for uplink users, and the average results from 30 experimental runs are plotted in the graph data points. The events happen randomly in the network at different places, which is considered over the simulation period of 1000 s.

### 5.2. Performance Metrics

The effectiveness of the proposed PAUM system has been demonstrated using the following performance metrics:

*Average Utility*: It measures how the high priority users with higher data rate receive transmission opportunities in the network. Utilities achieved by individual UEs, as calculated in Equation (4), are then averaged to plot the graph.

*Average Packet Delivery Ratio*: It is defined as the ratio of the number of packets delivered at an eNB to the total number of packets sent from all source nodes under that eNB. The higher value shows improved performance.

*Radio Resource Access Fairness*: This is used to determine whether users are receiving a fair share of system radio resources. Our system approaches Jain’s index, which provides a fairness criterion that considers all the users of the system, not only those assigned minimal resources. The fairness index starts from 0, and a value close to 1 indicates a fair share of radio resource access.

*Average Packet Delivery Delay*: It is the average time delay experienced by all data packets from UEs to reach at the corresponding eNB. The lower value indicates higher performance.

*Classification Accuracy*: It is a measurement of how correctly events are identified as critical, relevant, and normal after applying Bayesian learning.

### 5.3. Simulation Results

The obtained results from the simulation experiments using various aspects of different scenarios are discussed in this section. The simulation trace file is the subject of our in-depth investigation. RadioBearerStatCalculator from NS-3 is a trace sink that generates the number of transmitted PDUs (Protocol Data Unit), received PDUs, transmitted bytes, received bytes, and other PDU statistics for uplink users.

#### 5.3.1. Impacts of Varying Data Generation Rates from Sensor Devices

**Figure 4:**
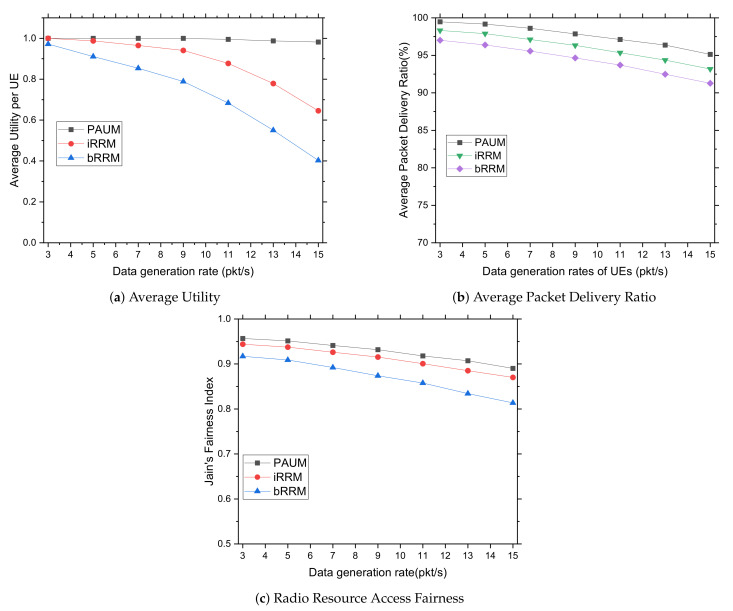
Impacts of varying data generation rates from sensor devices.

In this subsection, we study the performances of the radio resource management approaches for increasing data generation rates from the healthcare sensor devices. The rate at which data are generated (in packets per second) has a considerable impact on PAUM’s scalability and efficiency. This occurs as the traffic density is determined by the network applications and their traffic injection rates. In PAUM, the term utility refers to how well priority users are able to transmit data in a good channel with mitigated interference. The graphs in Figure 4a illustrate that, as a result of the network’s event-driven prioritization, the utility achieved by PAUM users remains stable while the data rate increases. It shows that the PAUM’s functionality is highly appreciable by the system’s users. In the case of PAUM’s prioritized event scheduling, the performances of average utility outperform over *bRRM* and *iRRM* because none of the research comes up with the utility measurement by well-managed resource allocation governed by higher priority with classified events. It is obvious that the *Utility* measurement of the PAUM is effective in terms of event prioritization as the rate of data generation increases. The graphs of Figure 4b show that as the number of packets generated per second increases, the average packet delivery ratio (PDR) slowly declines. The PAUM system achieves minimal data loss over *bRRM* and *iRRM* since it avoids congestion and allocates high-quality resources. Resource allocation is critical when it comes to assessing user usefulness. These findings support the use of PAUM for real-time healthcare applications leveraging NB-IoT. Finally, fairness ensures that resources are distributed to each user in accordance with their requirements. As illustrated in Figure 4c, the value of equitable resource sharing is maximized. It can be seen that because most schedulers focused solely on the channel conditions of UEs while allocating radio resources, their associated fairness increases as SINR values rise. However, this is not the case for the event-influenced prioritized *Multilevel Queue with Preemptive Round Robin scheduling scheme*, which maximizes the overall system utility by considering the UEs with best channel conditions along with their time slot; it automatically excludes all other UEs with relatively bad channel conditions, resulting in the highest fairness index. The PAUM outperforms *bRRM* and *iRRM* because it considers event type and priority, along with SINR value.

#### 5.3.2. Impacts of Varying Number of User Equipment (UEs)

**Figure 5:**
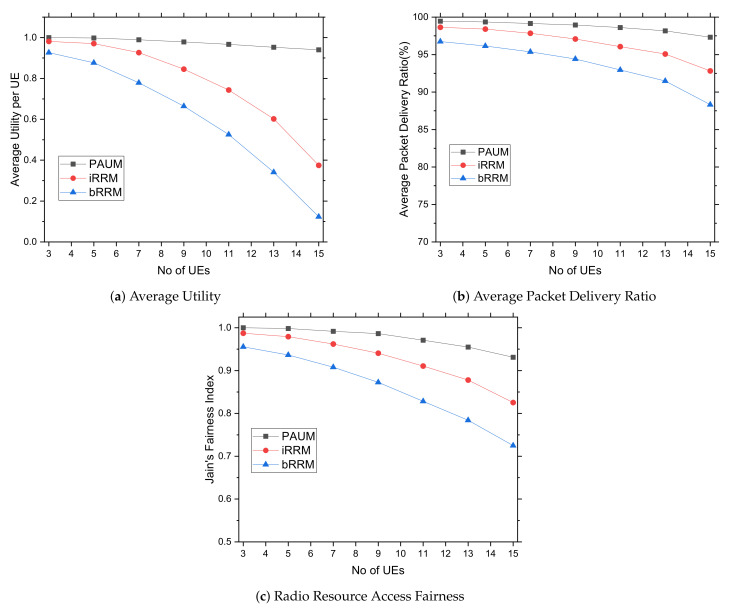
Impacts of varying number of User Equipment (UEs).

In this experiment, we choose the number of UEs randomly (with uniform distribution) from a range of 3–15 and the performance of varied numbers of UEs keeping the bandwidth, bit error rate, and the number of uplink channels at 66 kbps, $10^{-3}$ BER, and 2 with a 1 ms time slot, respectively. The high priority users having a higher data rate and less interference requirements are more valuable for data transmission than others, which is not addressed in *bRRM* and *iRRM*. As a result, the graphs in Figure 5a illustrate that in the case of PAUM, even a little increase in UE can maintain a steady utility for all UEs. Thus, the PAUM outperforms the *bRRM* and *iRRM* systems significantly due to smart user classification and prioritization.

The graphs in Figure 5b demonstrate that as the number of UEs grows, the average packet delivery ratio (PDR) decreases consistently for all examined approaches, indicating that the PAUM is effective for all classified events. It avoids interference and prioritizes UEs with higher PDR over UEs with lower PDR, such as *bRRM* and *iRRM*. It occurs because the priority-sorted event minimizes the average hop distance of delivered data packets. Despite the fact that *iRRM* and *bRRM* reduce interference while managing the radio resources, the packet delivery ratio of PAUM demonstrates the benefits of employing prioritized classified events, which outperforms both of them.

Finally, when compared to other systems, the proposed PAUM has the highest resource sharing fairness for changing numbers of UEs, as shown in Figure 5c. This is due to the network’s bandwidth being divided with suitable event urgency, using channelization on time by Equations (8) and (9). Due to a lack of priority and correct coordination with channelization and time slot with bandwidth, *bRRM* and *iRRM* do not adequately share resource allocation. The resources are equitably distributed across the UEs following their priorities, and the bandwidth is distributed in such a way that data transfer from the assigned node is reduced. As a result, PAUM achieved a higher fairness index over *bRRM* and *iRRM*.

#### 5.3.3. Delays Experienced by Different Packet Types vs. Data Generation Rate

**Figure 6:**
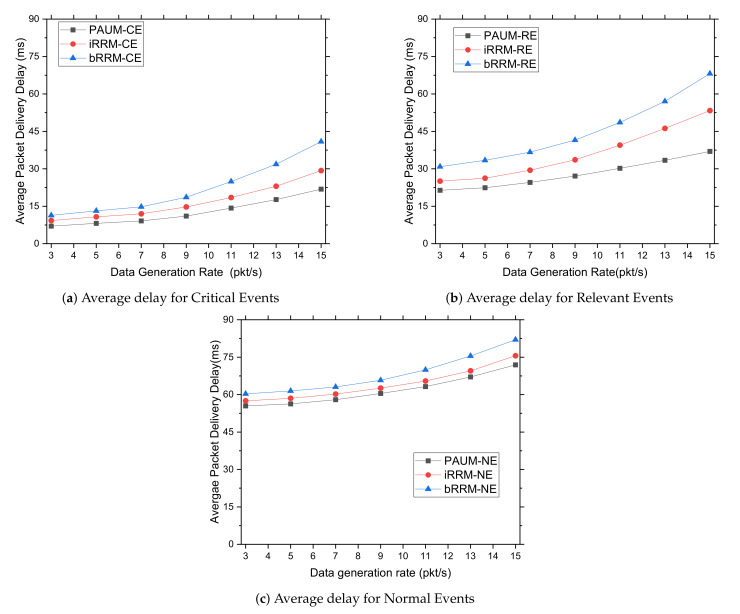
Delays experienced by different packet types vs. data generation rate.

This experiment investigates the effects of varying data generation rates on the average delays experienced by different data packet types. The *average packet delivery delay* constantly increases for all the studied systems with the increasing data generation rates, as illustrated in Figure 6. As a result of event-influenced prioritization, Critical Events (CE) experienced the least amount of delay compared to Relevant Events (RE) and Normal Events (NE), as expected theoretically. It is also revealed from the graphs of the Figure 6 that, on an average, the delays of the RE and NE packets are almost 1.5 times and 3 times higher, respectively, compared to the CE packets.

Such an excellent performance improvement is achieved due to the fact that, with the increasing data generation rates, the PAUM system efficiently categorizes different events and allocates radio resources in a prioritized manner and mitigating interference, whereas the generation of additional data packets cause resource allocation overhead to both the *iRRM* and *bRRM* systems since they do not differentiate among the data packets; rather, they try to allocate resources to all packets simultaneously, causing interference and congestion. Thus, the data delivery performances of the latter systems are degraded.

#### 5.3.4. Delays Experienced by Different Packet Types for Varying Number of UEs

**Figure 7:**
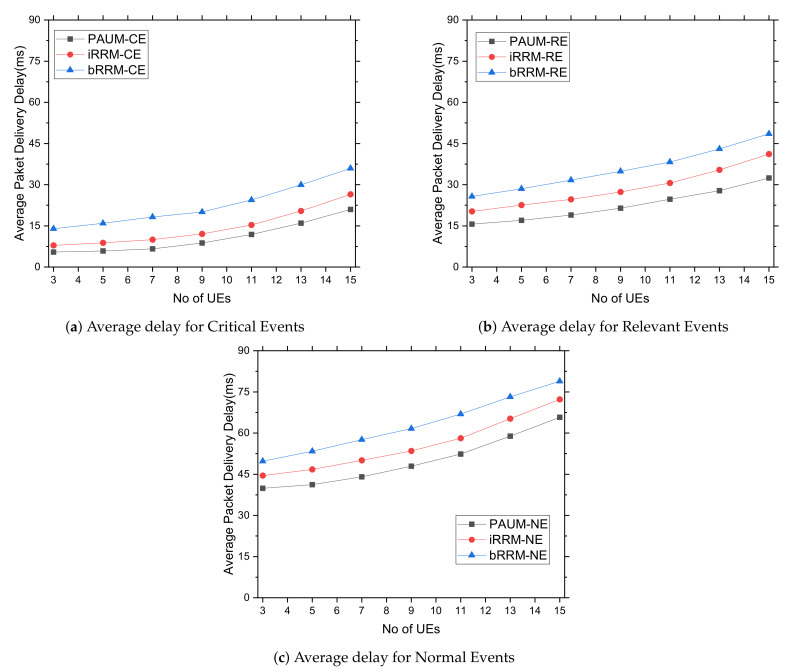
Delays Experienced by different packet types vs. User Equipment.

Next, we investigate the average data delivery delay experienced by different data packet types under an increased number of UEs, as shown in Figure 7. Since the increased number of UEs demands augmented transmission opportunities and injects more packets into the network, the general trend of increased delay is observed in all the studied cases. The *iRRM* considers the repetition factor, time offset, and data rate for radio resource management, which performs better than *bRRM* in terms of delay and which accounts for inter-carrier-interference only. Finally, the proposed PAUM system exhibits less delay compared to *bRRM* and *iRRM* for all data packets due to minimized interference and prioritized network access.

#### 5.3.5. Classification Accuracy

**Figure 8:**
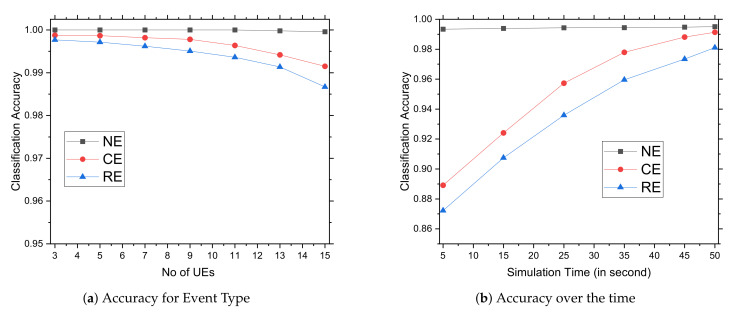
Classification Accuracy vs. User Equipment and Simulation Time.

The graphs in Figure 8a,b show classification accuracy of various event types exploiting the Bayes Theorem according to Algorithm 1. At the beginning, sensory data generated by the UEs are used as normal data for communication with eNB in an NB-IoT network. Subsequently, all collected data and their co-relations are used by eNB to classify the events. The eNB identifies event-wise training data, which serve as prior information for determining the likelihood of events, and then Bayesian learning assists in determining the probability of events using prior knowledge and associated conditions. The Figure 8a shows that as the number of UEs grow, the accuracy level gradually decreases. Note that when the number of UEs increases, many sensory inputs may not reach the eNB in a timely manner, causing the event relationships to be misinterpreted. If available data are not present, it can be difficult to locate relevant data for a comparable critical event; nevertheless, there is no trouble identifying a normal occurrence. As a result, regular events are identified with high accuracy, followed by critical events and relevant events. We did not compare classification accuracy with others because they do not have a classification system. Figure 8b depicts categorization accuracy as a function of simulation time, from 5 to 50 s. In the case of probabilistic determination, training data accumulate over time, allowing for more precise estimations of occurrence likelihood. As time passes, training data accumulate, resulting in a more accurate type of event and a more accurate event association. In this diagram, the accuracy for normal events has remained constant since the beginning, whereas critical and relevant event accuracies have been increased over time.

## 6. Conclusions

This work investigated how healthcare application events are influenced by priority access scheduling and utility maximization in NB-IoT. Our suggested system, PAUM, greatly contributed to resource allocation for prioritized access by allocating a suitable channel, a non-interfering time slot with greater data rate to different data packets by formulating a mixed non integer linear programming solution. Using event-induced prioritization, our system maximizes the utility value of users by allocating resources optimally. Our in-depth examination of the simulation trace file revealed that interference was reduced as a result of prioritized access scheduling to the time slots. Due to event classification, the critical event received the greatest priority to meet its urgency, followed by the other events. The simulation results showed significant performance improvement in terms of utility, fair share of resources, delay, and packet delivery ratio by 65%, 45%, 25%, and 15%, respectively, compared to the *iRRM* system.

In the future, in place of Bayesian learning, other learning approaches including instance-based algorithms, analytical learning, and reinforcement learning can be explored to study scopes for further performance improvement.

## Figures and Tables

**Table 1:** Notations.

Symbol	Meaning
*N*, *V*	Set of UEs and vital signs, respectively
*S*, *E*	Set of hypotheses and Events, respectively
$\partial_{C}$, $\partial_{R}$, $\partial_{N}$	Critical, Relevant, and Normal training data, respectively
ρ	Received power of a user
*h*	Channel gain
*I*	Interference
$N_{0}$	Additive White Gaussian Noise
$r_{z}$	Data rate of uplink user, z∈Z
*U*	User Utility

**Table 2:** Events Classification.

Disease	Probable Location	Event Description	Event Type
*Acute severe illness or life saving patient*	(i) Emergency (ii) Urgent Operation Theatre	(a) Respiratory rate, Heart Rate, Blood Sugar	(a) Critical Event
	(iii) Remote Location	(b) Blood Pressure, Oxygen Saturation, Chest X-Ray, Kidney, Liver Functionalities	(b) Relevant Event
*Asthma*	(i) Emergency (ii) Urgent Operation theatre	(a) Oxygen Saturation, Respiratory rate	(a) Critical Event
	(iii) OPD (iv) Cabin	(b) Heart Rate, Fever, Cough	(b) Relevant Event
*Acute Gastritis*	(i) Emergency	(a) Ultrasound, Endoscopy, Colonoscopy	(a) Critical Event
	(ii) OPD (iii) Cabin	(b) Food Habit, Stress Measurement	(b) Relevant Event
*Heart Disease*	(i) Emergency (ii) Urgent Operation Theatre	(a) Blood Pressure, Creatinine test	(a) Critical Event
	(iii) OPD, Cabin (iv) Remote Location	(b) Chest X-Ray, ECG, Echo	(b) Relevant Event
*Regular Test for Kidney, Liver*	(i) OPD (ii) Cabin (iii) Remote location	(a) Creatinine, Uric Acid, Ultrasound, Fever	a) Normal Event
*Regular Test for ENT*	(i) OPD (ii) Cabin (iii) Remote location	(a) X-Ray, Blood Test	(a) Normal Event
*Regular Test for EYE*	(i) OPD (ii) Cabin (iii) Remote location	(a) Opthalmology machine screening	(a) Normal Event
*Regular Test for Gynae and Obs*	(i) OPD (ii) Cabin (iii) Remote location	(a) Blood Pressure, Movement of fetus, Heart rate	(a) Normal Event

**Table 3:** Simulation Parameters.

Parameters	Values
Cellular layout	Hexagonal Grid, 3 sectors per site
Carrier	900 MHz
Inter-site distance	500 m/1732 m
UE deployment	Uniform random distribution
Event generation at UEs	Uniform random distribution
BS Transmit Power	43 dBm
UE transmit power	Maximum 23 dBm
Inter-site correlation co-efficient	0.5 and 0.75
ϵ	0.0005

## Data Availability

Not applicable.
